# *Coffea arabica* L. Resistant to Coffee Berry Borer (*Hypothenemus hampei*) Mediated by Expression of the *Bacillus thuringiensis* Cry10Aa Protein

**DOI:** 10.3389/fpls.2021.765292

**Published:** 2021-10-22

**Authors:** Eliana Valencia-Lozano, Jose Luis Cabrera-Ponce, Juan C. Noa-Carrazana, Jorge E. Ibarra

**Affiliations:** ^1^Laboratorio de Bioinsecticidas, Departamento de Biotecnología y Bioquímica, Unidad Irapuato, Centro de Investigación y Estudios Avanzados del Instituto Politécnico Nacional (CINVESTAV), Irapuato, Mexico; ^2^Laboratorio de Transformacion Genetica de Plantas, Departamento de Ingeniería Genética, Unidad Irapuato, Centro de Investigación y Estudios Avanzados del Instituto Politécnico Nacional (CINVESTAV), Irapuato, Mexico; ^3^Instituto de Biotecnología y Ecología Aplicada (INBIOTECA), Universidad Veracruzana, Xalapa, Mexico

**Keywords:** Cry10Aa, *Bacillus thuringiensis*, coffee transformation, coffee berry borer, somatic embryogenesis

## Abstract

*Coffea* spp. are tropical plants used for brewing beverages from roasted and grounded seeds, the favorite drink in the world. It is the most important commercial crop plant and the second most valuable international commodity after oil. Global coffee trade relies on two *Coffea* species: *C. arabica* L. (arabica coffee) comprising 60% and *C. canephora* (robusta) comprising the remaining 40%. Arabica coffee has lower productivity and better market price than robusta. Arabica coffee is threatened by disease (i.e., coffee leaf rust), pests [i.e., *Hypothenemus hampei* or coffee berry borer (CBB) and nematodes], and susceptibility to climate change (i.e., drought and aluminum toxicity). Plant biotechnology by means of tissue culture inducing somatic embryogenesis (SE) process, genetic transformation, and genome editing are tools that can help to solve, at least partially, these problems. This work is the continuation of a protocol developed for stable genetic transformation and successful plant regeneration of arabica coffee trees expressing the *Bacillus thuringiensis* (Bt) toxin Cry10Aa to induce CBB resistance. A highly SE line with a high rate of cell division and conversion to plants with 8-month plant regeneration period was produced. To validate this capability, gene expression analysis of master regulators of SE, such as BABY BOOM (BBM), FUS3, and LEC1, embryo development, such as EMB2757, and cell cycle progression, such as ETG1 and MCM4, were analyzed during induction and propagation of non-competent and highly competent embryogenic lines. The particle bombardment technique was used to generate stable transgenic lines after 3 months under selection using hygromycin as selectable marker, and 1 month in plant regeneration. Transgenic trees developed fruits after 2 years and demonstrated expression of the Bt toxin ranging from 3.25 to 13.88 μg/g fresh tissue. Bioassays with transgenic fruits on CBB first instar larvae and adults induced mortalities between 85 and 100% after 10 days. In addition, transgenic fruits showed a seed damage lower than 9% compared to 100% of control fruits and adult mortality. This is the first report on stable transformation and expression of the Cry10Aa protein in coffee plants with the potential to control CBB.

## Introduction

Coffee (*Coffea arabica* L. and *C. canephora* Pierre ex A. Froehner) is the most valuable tropical export crop in the world, with an annual global coffee production of around 7.7 million tons of golden coffee, in an area of 10.5 million hectares in more than 50 countries (International Coffee Organization, 2019).

This production has been registered in spite of the susceptibility of *C. arabica* to diseases and insect pests, being the coffee berry borer (CBB), *Hypothenemus hampei* (Ferrari) (Coleoptera: Curculionidae: Scolytinae), one of the main pests in the world. CBB larvae in coffee seeds cause yield losses up to 80%, equivalent to US$500 million annually and affecting more than 25 million rural households involved in coffee production worldwide, due to reduction in grain weight, lower product quality (Moore and Prior, 1988; Vega et al., 2003), premature fruit fall due to early infestations (Schmitz and Crisinel, 1957; Kraker, 1988; Mairena-Ortíz et al., 1991), and physical damage to the fruit, which allows infestation and attacks by other pests (Leefmans, 1923; Penados and Ochoa, 1979).

Coffee berry borer control depends on the use of chemicals (i.e., endosulfan) and some biological agents, such as bethylid parasitoids (*Cephalonomia stephanoderis* and *Prorops nasuta*) and entomopathogenic fungi (*Beauveria bassiana* and *Metarhizium anisopliae*). These agents show their greater efficiency on the surface of the berry, having an effect mainly on adults (Gingerich et al., 1996; Bustillo et al., 1998).

*Bacillus thuringiensis* (Bt) has contributed globally to insect pest control since the 1960s (Heimpel and Angus, 1960). Currently, more than 800 Cry protein sequences have been recorded, grouped into 78 different classes, and are specifically active mainly against insects and nematodes (Crickmore et al., 1998). The optimized nucleotide sequences, which code for the active fragment of the Cry proteins of Bt in plants, have been successfully cloned, integrated, and expressed through the use of genetic engineering in different crops.

The Bt-protected crops, such as corn, cotton, soybeans, and potatoes, have shown significant benefits since their introduction in 1996. These materials provide a level of protection against insects that is generally higher as compared to conventional chemical pesticides. As a result, Bt crops require fewer synthetic pesticide applications (if any), which avoid exposure to toxic compounds; in addition, they help to preserve the population of beneficial insects, such as parasitoids and predators (Klotz-Ingram et al., 1999; Betz et al., 2000).

Transgenic coffee plants expressing Cry proteins were first developed by Leroy et al. (2000) and evaluated under field conditions by Perthuis et al. (2015). These plants express the Cry1Ac protein that confers resistance to the coffee leaf miner, *Leucoptera coffeella* (Lepidoptera: Lyonetiidae), with successful damage inhibition. Most of the characterized Cry toxins are active against Lepidoptera, and to a lower extent, to coleopteran species (De Maagd et al., 2001; James et al., 2009; Shah et al., 2016). Méndez-López et al. (2003) demonstrated that *B. thuringiensis* serovar *israelensis* (Bti), which contains the Cry10Aa protein at very low expression levels, exhibits high toxic levels against CBB. Subsequently, the specific and efficient activity of Cry10Aa was demonstrated *in vitro* toward the cotton boll weevil, *Anthonomus grandis* Boheman (Coleoptera: Curculionidae) (de Souza-Aguiar et al., 2012) and in transgenic cotton plants under greenhouse conditions, showing high levels of toxicity against *Anthonomus grandis* (Ribeiro et al., 2017).

Most genetic transformation protocols are based on the integration of the genes of interest into the plant genome in undifferentiated plant tissues, such as somatic embryos (SEMs). However, arabica coffee varieties have been demonstrated to be more difficult to transform than robusta coffee.

Long-term and fast-growing SE with a high rate of plant conversion is a very important requirement to obtain stable transgenic trees and seed development. The induction and maturation of *C. arabica* var. Typica SEMs were generated under osmotic stress conditions (Valencia-Lozano et al., 2019, 2021). Highly competent (HC) embryogenic lines were generated and used for genetic transformation with Bt *cry10A* toxin coding gene.

To understand basic molecular mechanisms affecting SE in *C. arabica* and genetic transformation competence, genes involved in SE, such as BABY BOOM (BBM), FUS3, and LEC1; embryo development, such as EMB2757; and cell cycle progression, such as ETG1 and MCM4, were analyzed during induction and propagation of non-competent (NC) and HC embryogenic lines.

In this study, we followed an efficient and reliable protocol for genetic transformation of *C. arabica* var. Typica. Fruits from transgenic trees showed expression of the Bt toxin. Bioassays with transgenic fruits induced high CBB mortality and very low seed damage. Stable transformation and expression of the Cry10Aa protein in coffee plants show potential to control the CBB.

## Materials and Methods

### SE Induction of *C. arabica* Var. Typica

Plant material was obtained from the protocol developed by Valencia-Lozano et al. (2019). SE of *C. arabica* var. Typica was induced from leaf explants collected from 8-month-old trees in Tapachula, Chiapas, Mexico. Explants were superficially disinfected according to Cabrera-Ponce et al. (2015), and the protocol for induction and propagation of SEMs was adapted from traditional medium by Van Boxtel and Berthouly (1996) and modified CP2 medium by Valencia-Lozano et al. (2019). The SE lines derived from *C. arabica* var. Typica after 2 months of subculture in globular stage on traditional and modified CP2 media, 24-h mannitol 0.15-M–Sorbitol 0.15-M treatment before and after bombardment, were used.

### Gene Expression Analysis of SEMs Used for Genetic Transformation

To select genes involved in the SE development process, a gene network with a high confidence (0.700) was performed with the software STRING v11.0 (http://string-db.org; Szklarczyk et al., 2019) based on *C. arabica* homologous genes present in the *Arabidopsis thaliana* genome.

The selected genes were those involved as gene master regulators of SE (BBM, FUS3, and LEC1), embryo development (EMB2757), and cell cycle (ETG1 and MCM4). These genes were analyzed during the induction and propagation process.

Gene identifier was made according to UniProt (http://www.uniprot.org), NCBI (http://www.ncbi.nlm.nih.gov), and Phytozome database (Goodstein et al., 2012). Sequences of all genes were analyzed from *A. thaliana* using blastN and blastP in the coffee genome homologous sequences.

Homologous sequences in *C. arabica* genome >40% in protein sequence with *A. thaliana* were considered. Proteins of *C. arabica* homologous with *A. thaliana* aligned by blastP were identified as: XP_027062561 (BBM), XP_027089900.1 (EMB2757), XP_027110645.1 (ETG1), XP_027102113.1 (FUS3), XP_027085797 (LEC1), and XP_027112176.1 (MCM4) (Table 1). Oligonucleotides were designed (Table 1) to use them in qPCR (2^−ΔΔCT^) analysis.

**Table 1:** Primers designed to be used in qPCR analysis of selected genes.

***Arabidopsis thaliana***	***Coffea arabica***	**Query cover**	**Ident**	**Forward**	**Reverse**
LEC1	XP_027085797	46%	74.77%	CCAGGAATGTGTATCGGAGTAC	GAAAGCGGTGGAGATATAGGG
BBM	XP_027062561	67%	56.00%	TTCAACCCCAACGAGATCAG	GTTGTAGTTCTCCTTCCAGTCC
FUS3	XP_027102113.1	85%	44.44%	GGCTTACGACATGGAGACTAC	GCATTTATCTCCGACTCAGGG
EMB2757	XP_027089900.1	99%	59.53%	CCATCCCTCCAAGAACATACTG	CTCCAATCAAAGGACTAGCCG
ETG1	XP_027110645.1	97%	59.01%	GTGCCTCGTATCCATTGTCTAG	CCGTCATTTCCAAGAACAGC
MCM4	XP_027112176.1	100%	74.77%	ACCCTATCCAGCACAAATCC	ACGCTTATGTTAGTTCCCCAG
RPL39	XM_027221555.1	100%	99.66%	GCGAAGAAGCAGAGGCAGAA	TTGGCATTGTAGCGGATGGT
24S	XM_027261007.1	100%	99.12%	GACCAATCGTCTTCTTTCCAGAAA	TCAACTCAGCCTTGGAAACATTAG
ACT	XM_027221492.1	99%	99.44%	GCCAGATGGACAAGTGATTACCA	CAGCAGCTTCCATTCCTATGATAG

### RNA Isolation and qPCR Analysis

Total RNA from SEMs cultured in traditional medium (NC) and modified (CP2) medium (HC) was isolated using TRIzol (Invitrogen, Carlsbad, CA, USA) (UniProt Consortium, 2014). RNA concentration was measured by its absorbance at 260 nm (ratio 260 nm/280 nm was assessed), and its integrity confirmed by electrophoresis in agarose 2% (w/v) gels.

The cDNA samples were amplified by PCR using the SYBR Green qPCR System (Bio-Rad, Hercules, CA, USA) in Real-Time PCR Systems (CFX96 Bio-Rad). The reference genes in this work were ACT, 24S, and RPL39, according to Freitas et al. (2017) applied for the qPCR analysis of coffee SEMs.

Retfinder, NormFinder, Bestkeeper, and Delta-Ct were used in this analysis by triplicate. Relative expression was calculated, and weighted ct, and next, a delta ct in each gene was analyzed and relative amount of target gene expression using the 2^−ΔΔCT^ method (Livak et al., 2013). The qPCR analysis was based on at least three biological replicates for each sample with three technical replicates and control treatment.

### Greenhouse Establishment of Transgenic Tress and Fruit Development

The SEMs maturation and plant regeneration of transgenic and wild-type SEMs were made according to Valencia-Lozano et al. (2021). Three transgenic events and wild-type plants were successfully acclimatized for 8 months in growth chambers. Transgenic and wild-type plants were transferred to experimental greenhouses at the IMBIOTECA (Xalapa, Veracruz, Mexico) and developed under controlled agroclimatic conditions (relative humidity >75% and temperature between 22 ± 2°C).

Flowering occurred after 12 months, and fruits were evaluated 125 days after anthesis until they showed 20% dry matter. These are the optimal conditions for the CBB infestation, required to check for the efficiency in the control of *H. hampei* in the transformed plants.

Transgenic fruits showed similar dimensions, within the range of commercial arabic varieties (e.g., Var. Typica and Var. Colombia) and comparable with the non-transformed wild type.

### Southern Blot Hybridization Analysis

Total genomic DNA was isolated from fruits of three transgenic tree lines established under greenhouse conditions and including wild-type plants, as described by Valencia-Lozano et al. (2019). Aliquots of 20 μg of genomic DNA were digested with *Eco*RI and *Hin*dIII (10 U/μg each), which fragmented the *cry10Aa* sequence into three bands (963, 598, and 410 bp). Products were then electrophoresed in a 1.0% agarose gel and transferred to positively charged nylon membranes (Hybond-N+, Amersham, Little Chalfont, UK) using 2× SC, as described earlier (Sambrook et al., 1989). Membranes were prehybridized for 24 h at 60°C in 2 × SCP, 0.5% BSA, and hybridized overnight at 60°C (Sambrook et al., 1989). The hybridized biotin-labeled probes were detected with streptavidin antibody conjugated with alkaline phosphatase (AP) and revealed using an AP Conjugate Substrate Kit (Thermo Fisher, Vilnius, Lithuania).

### Insect Rearing

**Figure 1:**
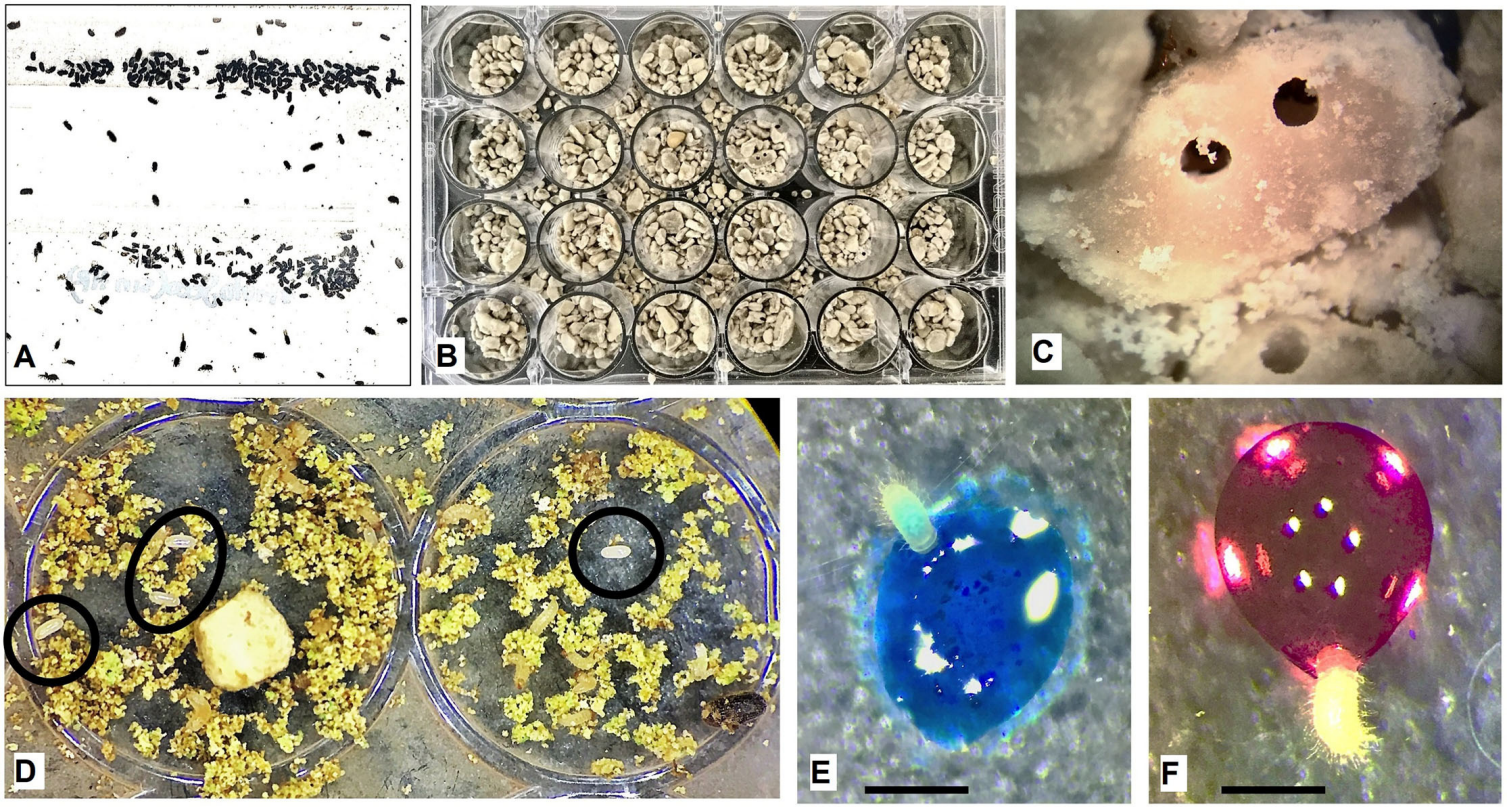
Bioassay using first instar larvae of *Hypothenemus hampei*. **(A)** Females (1.6–1.9 mm long) recovered from a laboratory colony. **(B)** Individualization of *H. hampei* females in 24-well titration dishes. **(C)** Close-up of coffee berry borer (CBB) drills in green coffee seed pieces (0.7 mm). **(D)** First instar larvae (0.019 mm) and egg (0.001 mm) after 20 days of infestation. **(E)** First instar larvae fed with 1 μl of transgenic plant extracts. **(F)** First instar larvae fed with 1 μl of control plant extract. Bars: 0.5 mm.

To demonstrate the insecticidal efficiency of the *cry10Aa* gene in transgenic coffee plants, a laboratory colony of *H. hampei* was established from individuals collected in coffee fields (Xalapa, Veracruz, Mexico), and females were recovered (flying and walking) from each fruit (Figure 1A). As known, sibling male and female mate within the berry; males die, and the fertilized females leave the berry to find a new berry in which to deposit their eggs (Baker et al., 1992).

Females were isolated in humid chambers, disinfected with 1.2 g/L of Benomyl (Bustillo Pardey, 2006), and incubated at >75% RH, 8/16 h photoperiod, and temperature of 22 ± 3°C until complete the cycle (30 days) to have a homogeneous population. F1 females obtained under laboratory conditions were transferred to 24- and 96-well titration plates on green coffee seed pieces. Eggs were selected for the bioassay after 15 ± 5 days (Figures 1B–D).

### Bioassays With Protein Extracts From SEMs and Leaves

Proteins extracted as described earlier (Valencia-Lozano et al., 2019) from three SEMs and three-leaf samples of transgenic lines, showing differential expression of the Cry10Aa among them, were quantified by ELISA. The six concentrations obtained were reported by Valencia-Lozano et al. (2019) corresponding to events 1, 2, and 3 (Table 2).

**Table 2:** Cry 10Aa concentrations found in SEMs and leaves from three transformation events of coffee.

**Transgenic line**	**Clone**	**SEMs (μg/g fresh weight)**	**Leaves (μg/g fresh weight)**
Event 1	6	13.88	8.67
Event 2	8	12.7	7.8
Event 3	9	4.6	3.25

Each first instar larva (2 days after hatching) was fed individually with 1 μl of total protein extract (200 mg/ml) dissolved in water with 20 μl of food dye to observe the intake of the toxin (Figure 1E). Negative control was fed with water and food dye (Figure 1F). All food dyes (red, blue, green, and yellow) were previously tested negative for any toxic effect. After 2 h, larvae were washed with distilled water to remove surface dye residues and transferred to 24-well plates, with green coffee seed pieces, under dark conditions at 25°C and 70% RH. Each experiment was performed in triplicate for a total of 60 individuals per dose. The toxic activity was evaluated daily for 10 days.

The Cry10Aa concentrations found in each transformation event were used as a dose to carry out a preliminary Probit analysis for the estimation of an LC_50_ (concentration that generates 50% mortality in test insects), as a statistical parameter that reflects the level of toxicity (Ibarra and Federici, 1987). A regression line was established between the Cry10Aa concentrations and the mortality percentages, both transformed to logarithms and Probit units, respectively. The main estimated parameters were: the slope, the chi-square value, and LC_50_.

### Bioassay With Transgenic Coffee *C. arabica* Fruits

Fruits harvested 125 days after flowering from three transgenic events of coffee were inoculated with CBB-fertilized females. The bioassay was made in closed plastic containers, where each fruit was infested with a female and incubated in a growth chamber at 25°C and RH 70%.

Female penetration to fruits, mortality, and emergence of new adults were evaluated daily. When the emergence of the first adults in control fruits was observed, a cross-section was made in all tested fruits and percent damage, development of CBB larvae, and resistance to fruits by *H. hampei* infestation was quantified.

## Results

### Gene Expression Analysis of SEMs Used for Genetic Transformation

Understanding the basic molecular mechanisms affecting SE in *C. arabica* is keys to establish genetic transformation protocols, based on the integration of the genes of interest into the plant genome in undifferentiated plant tissues, such as SEMs. A line with high SE capabilities and high cell division rate, followed by the conversion into plants in 8-month, was generated under osmotic stress conditions. This HC embryogenic lines were generated and used for genetic transformation with the *cry10A* gene, which showed efficient expression of the Bt toxin.

**Figure 2:**
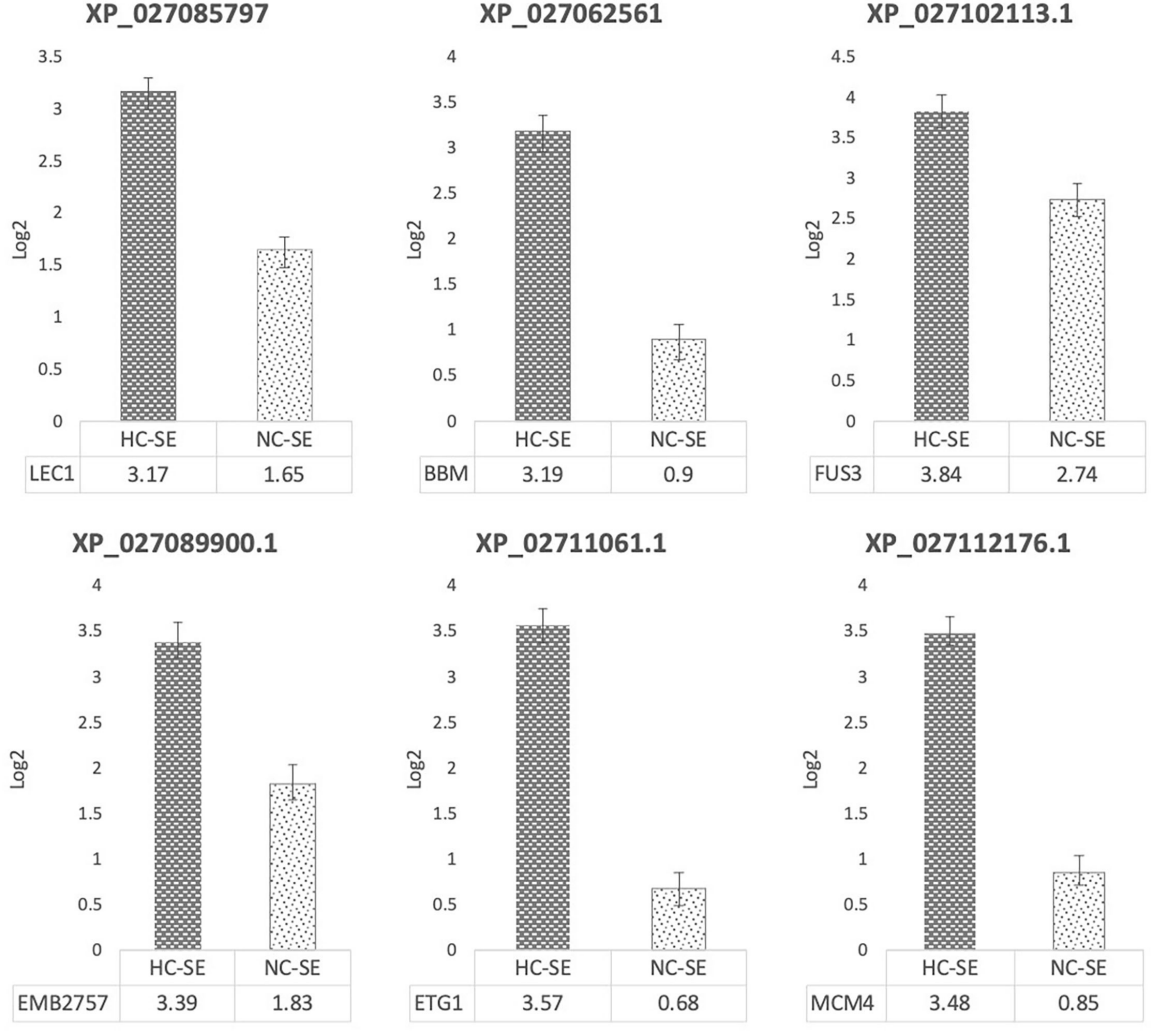
Gene expression analysis by qPCR during SE induction in highly competent embryogenic line (HC-SE) and non-competent line (NC-SE). Relative expression levels were plotted based on Log2 values, normalized with RPL39 (ribosomal protein L39), ACT (β-actin), and 24S (Ribosomal protein 24S).

To understand the molecular mechanisms involved in SEMs development and to validate this capability, a STRING-based bioinformatic analysis with high confidence (0.700) based on *C. arabica* homologous sequences in the *A. thaliana* genome was performed. As expected, upregulation during induction and propagation of embryogenic lines, cultured in traditional medium (NC) and modified (CP2) medium (HC), was found in genes involved in SE: BABY-BOOM (XP_027062561), FUS3 (XP_027102113.1), and LEC1(XP_027085797), embryo development EMB2757 (XP_027089900.1), and cell cycle progression ETG1 (XP_02711061.1) and MCM4 (XP_027112176.1) (Figure 2).

As expected, the expression level of LEC1, a nuclear transcription factor Y subunit B-9, was upregulated 3.17 times in the HC-SE line as compared to the regulated expression in the control of 1.65 times in NC-SE line. BBM, an AP2-like ethylene-responsive transcription factor, was upregulated 3.19 times in HC-SE line as compared with the control of 0.9 times in NC-SE. FUS3, a regulator of gene expression during late embryogenesis, was upregulated 3.84 times in HC-SE line, as compared to the control of 2.74 in NC-SE line. EMB2757/TAIMEN, which encodes a WD repeat protein with seven WD repeat motifs, was upregulated 3.39 times in HC-SE line, as compared to the control of 1.83 in NC-SE line. ETG1, an associated component of the mini-chromosome maintenance complex-binding protein that acts as a regulator of DNA replication, was also upregulated 3.57 times in HC-SE line, as compared to the control of 0.68 in NC-SE line. MCM4 acts as component of the MCM2-7 complex (MCM complex), which is the putative replicative helicase essential for DNA replication initiation and elongation, and was upregulated 3.48 times in HC-SE line, as compared in the control of 0.85 times in NC-SE line (Figure 2).

### Greenhouse Establishment of Transgenic Tress and Fruit Development

**Figure 3:**
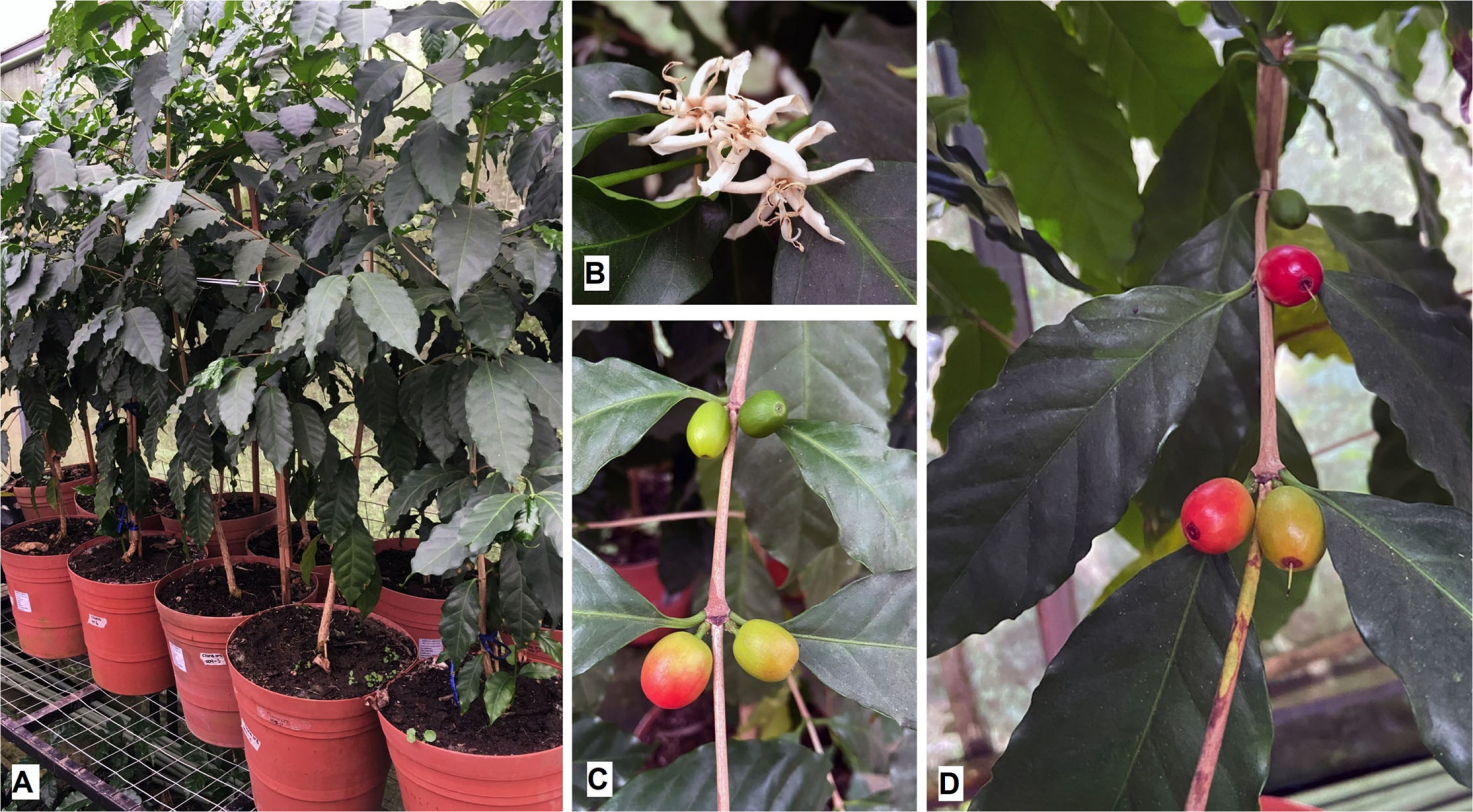
**(A)** Transgenic *Coffea arabica* plants grown under greenhouse conditions after 24 months. **(B)** Flowering of transgenic coffee trees after 18 months under greenhouse conditions. **(C,D)** Fruit development from transgenic coffee plants after 22 months under greenhouse conditions.

Transgenic and wild-type plants regenerated according to Valencia-Lozano et al. (2021) developed robust plants with a prominent root and leaf area (Figure 3A), and the length of the internodes. Flowering and seed and fruit production occurred after 12 months (Figure 3B). Fruits were evaluated until day 125 after anthesis (Figures 3C,D), when they showed 20% dry matter. These are the optimal conditions for CBB infestation, and therefore the optimal time to perform the final check of the efficiency in the control of *H. hampei* in the transformed plants. Fruits were selected from three different clones corresponding to three independent transformation events with similar dimensions within the range of commercial arabic varieties and comparable with the non-transformed wild type.

### Southern Blot and Hybridization Analysis

Fruits from three different transgenic events grown under greenhouse conditions were analyzed by Southern blot hybridization analysis to confirm the sexual segregation of *cry10Aa* gene in *C. arabica* var. Typica.

**Figure 4:**
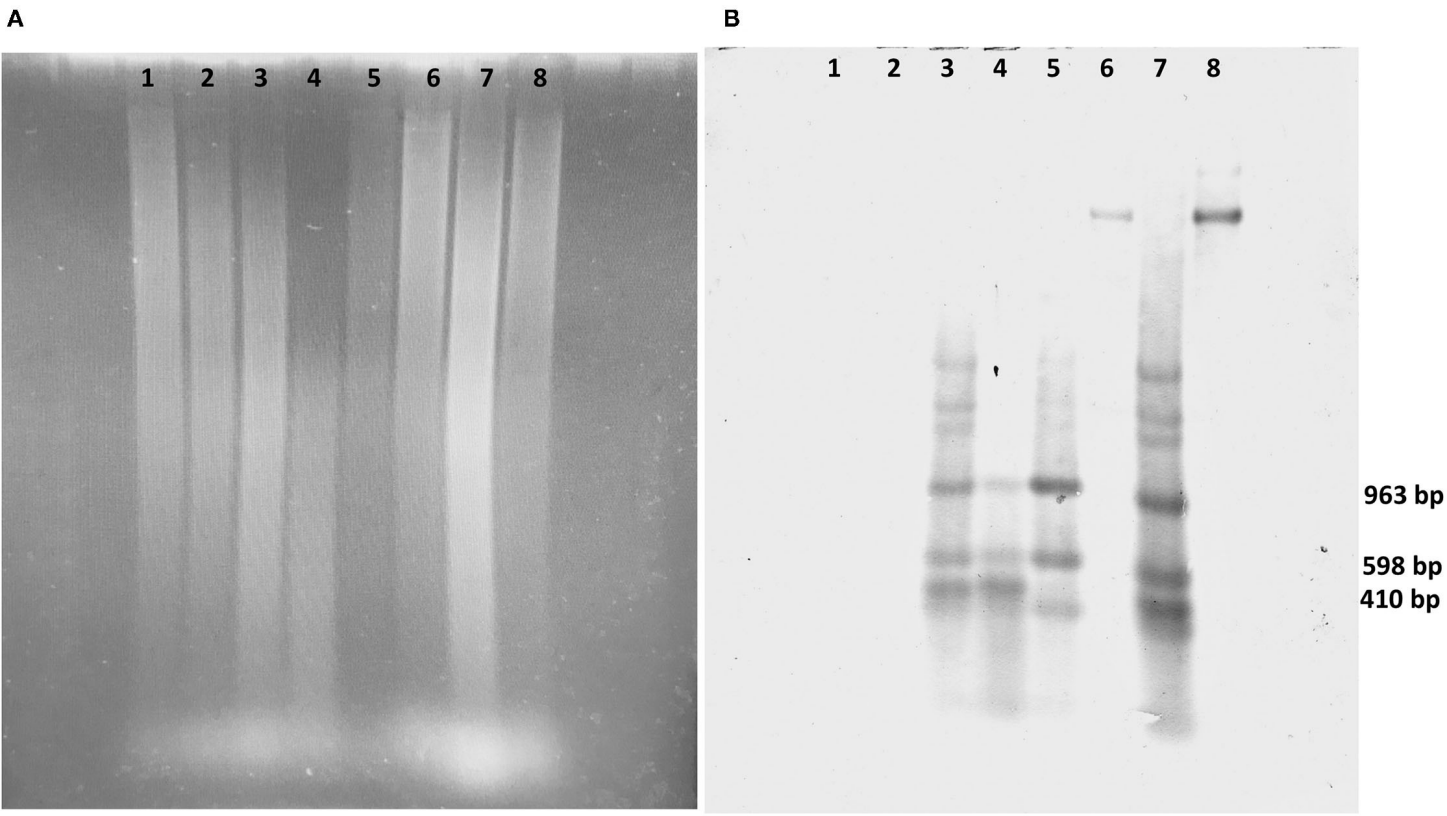
Southern blot and hybridization analysis of transgenic coffee fruits. **(A)** Electrophoresis of DNA digest of transgenic fruits and SEM of *Coffea arabica* digested with *Eco*R1/*Hin*dIII. **(B)** Southern blot analysis hybridized with *cry10Aa*-biotin of 1,993 bp probes. Lane 1, DNA from wild-type fruits; lane 2, wild-type SEMs; lane 3, transgenic fruits, event E1; lane 4, transgenic SEM event E1; lane 5, transgenic fruits, event E2; lane 6, partially digested transgenic SEM, event E2; lane 7, transgenic fruits, event E3; lane 8, partially digested transgenic SEM, event E3.

Expected hybridization signals of 963, 598, and 410 bp, according to the *cry10Aa* gene sequence digested *Eco*RI/*Hin*dIII, were observed in all transgenic events. The presence of positive hybridization signals of the predicted size showed that gene integration and sexual segregation of at least one foreign gene copy (*cry10Aa*) occurred in coffee plants derived from our highly competent SE line. No signal was detected in wild-type plants (Figure 4).

Tested transgenic clones displayed unique hybridization patterns, indicating that these transgenic plants were derived from independent transformation events. In all transgenic lines, bands with molecular weights different from expected were found. This indicates either that multiple independent insertions occurred, or that the integrated fragments are long tandem repeats resulting from re-arrangements.

### Toxicity and Mortality of CBB *H. hampei* With Extracts From Transgenic SEMs and Leaves

**Figure 5:**
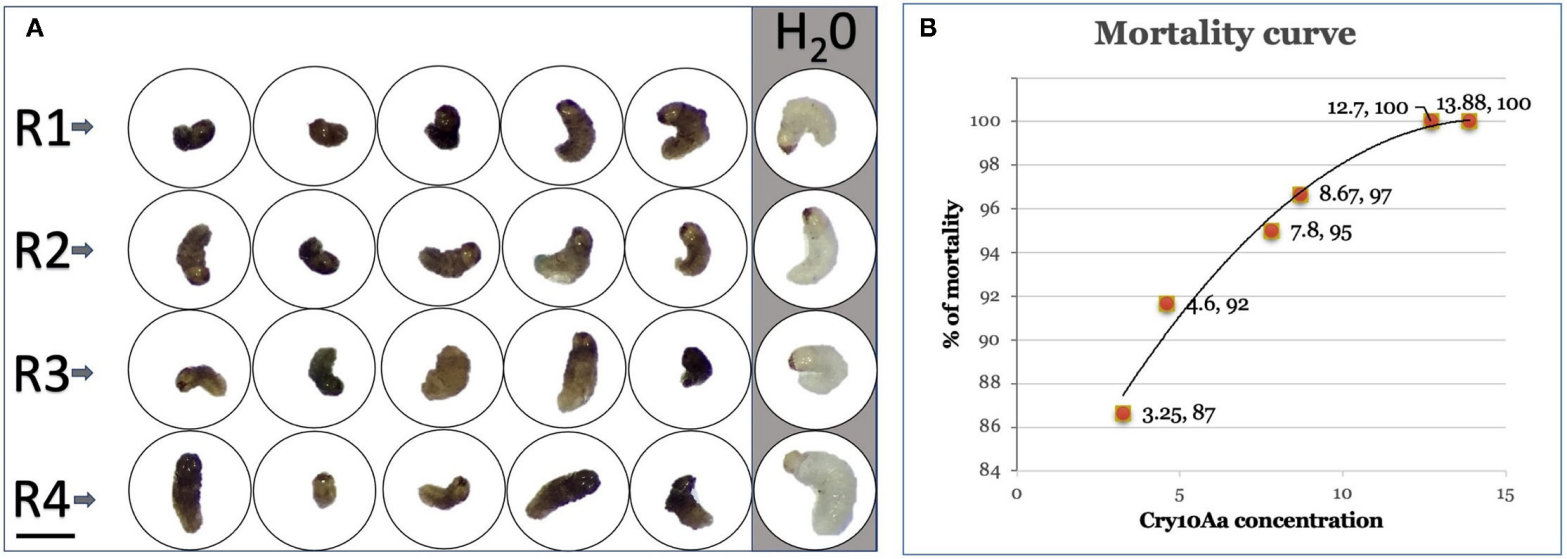
Mortality of coffee berry borer (CBB) first instar larvae. **(A)** Average morphology of first instar larvae in 24-well titration plates at 10 days. R1, R2, R3, and R4: Replicates of bioassays with larvae fed with Cry10Aa extract. Vertical gray line, water-fed larvae. Bar: 1 mm. **(B)** Mortality curve for Cry 10Aa concentrations found in SEMs and leaves from three transformation events of coffee (μg/g fresh weight).

The CBB bioassays were evaluated with six different concentrations of the toxin Cry10Aa quantified by ELISA from extracts (total protein) from leaves of transformed plants and embryogenic lines, using first instar larvae of *H. hampei*. Toxic activity of Cry10Aa against larvae of *H. hampei* began with mobility loss and turgor change and ended up showing body darkening after 2–5 days due to the onset of septicemia (Figure 5A).

Mortality of CBB was observed after 24 h in the higher concentrations (25%), reaching 96% at day 7. After 10 days, mortality raised to 87% with a Cry10Aa concentration of 3.25 μg/g fresh weight, 92% with 4.6 μg/g, 95% with 7.8 μg/g, 97% with 8.67 μg/g, and 100% with 12.7 and 13.88 μg/g (Figure 5B).

It should be noticed that CBB larvae normally show a living range of 20–26 days; however, and in the bioassays, we observed mortalities of 100% in the middle of that period. In addition, the amount of expressed Cry10Aa guarantees a 25% immediate control (24 h), and in a period of 10 days, total control is achieved inside the fruit in transformed coffee plants (Figure 5).

### Probit Analysis

As the quantification of expressed Cry10Aa varied from 3.25 to 13.88 μg/g fresh weight among the different transformation events, a relationship dose/mortality was analyzed by Probit analysis to estimate a preliminary LC_50_ for transgenically expressed Cry10Aa. The chi-square value was estimated at 2.34 (lower than the number of doses tested), the slope was 2.39 (it should be between 1.5 and 6), the LC_50_ was 1.202 (0.56 ± 2.56) μg/g, and the LC_95_ was 5.82 (4.55 ± 7.44) μg/fresh weight. The natural mortality in the negative control was 1.6%, which is within the recommended limits.

### Bioassays Against Adults Using Transgenic Fruits

The lethal effect of the transgenically expressed Cry10Aa protein on *H. hampei* was evaluated, starting with the infestation of transgenic fruits with CBB adults, during a period of 30 days, a period in which the emergence of the first adults of the next generation was observed in control fruits.

We quantified four steps of colonization levels by the female within the berry: (1) the female initiated the penetration of the exocarp before dying; (2) the female penetrated the endocarp, and only part of the abdomen is visible before dying; (3) the female is no longer visible and has bored into the endosperm where it died; and (4) the female constructed galleries and oviposited within the seed, where emerging larvae eventually died.

**Figure 6:**
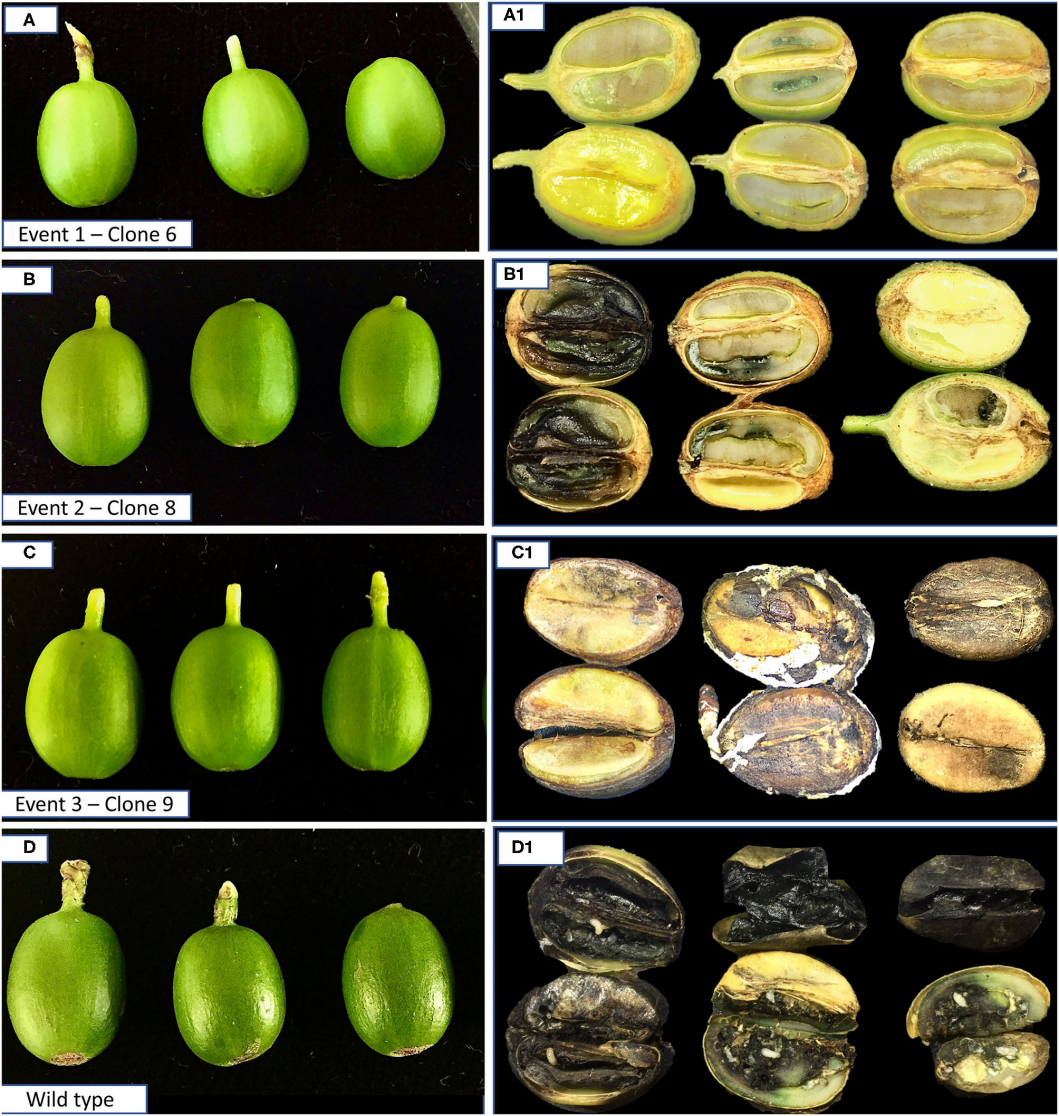
Fruits 125 days after anthesis from Cry10Aa transgenic plants infested with coffee berry borer (CBB) adults. Three transformation events. Events 1, 2, and 3 and a wild-type plant **(A–D)** were selected for the performance of bioassays. At the right-hand side, the evaluation of damage by cross section of fruits of F1 in the three events and the wild-type fruits after 30 days.

Each fruit was individualized in a container with a fertilized (flying) female. In the first 24 h, 100% of control fruits were perforated, and 67% of clone 8 (Event 2) (Figure 6). About 67% of fruits of clone 9 (Event 3) were perforated 48 h later, and fruits from clone 6 (Event 1), only 33% were partially perforated until after 20 days.

Scrapings on the exocarp near the micropyle and excrement were observed on the fruit, and on dead females, which indicates that the insect fed on the fruit, but was unsuccessful to penetrate. In 67% of fruits of this clone, the average female survival was 5 days, but after day 20 the rest of the females were able to drill the fruit but unable to penetrate it.

Fruits from clone 8 (Event 2) showed perforations in the peduncle and in the micropyle; however, 50% of females were dead on day 10. These qualitative evaluations indicated the activity of adults within the fruit from day 1 to day 10, shown by the presence of excrement outside the micropyle. This was more evident in the wild-type fruits, whereas some other events showed fewer excrement such as in clone 9 (Event 3) and clone 8 (Event 2), but a complete absence was detected in fruits from clone 6 (Event 1). Surveillance kept going until day 30, when the emergence of the first adults was observed in the control fruits. No adults emerged from any of the transgenic fruits. At this point, fruits were cross-sectioned to evaluate larval survival and levels of infestations (Figure 6).

About clone 6, female penetration in the transformed fruits was <12%. Some adults were able to drill to the interior of the fruit, causing no damage. In no case, larvae were found in transgenic fruits. In 33% of fruits (Figure 6), the presence of an egg was observed for a period of 10 days, but most females died, and no damage was detected on the seed. This clone showed the highest expression level of the Cry10Aa toxin.

Conversely, 70% of clone 8 fruits showed boring of multiple entrances and exits of females, with an average of only three adults, and two pupae, within a period of 30 days. Seed damage was much lower than that shown by the control fruits (Figure 6D).

About 30% of seeds showed saprophytic damage due to the penetration of bacteria into the galleries, but the presence of progeny development stages was not observed, and the female was dead (Figure 6B1)

Clone 9 presented contamination by fungus. Although the CBB could survive under these conditions, no progeny development was observed, nor was the fruit damage through galleries (Figure 6C1).

The results mentioned above demonstrate the toxic activity of the Cry10Aa protein on the adults and first instar larvae. Given the damage observed in the fruits of clone 8 and the absence of larvae, it could be attributed to a toxic activity directly to the infesting adults or to first instar larvae in the first days of the evaluation.

## Discussion

### Gene Expression Analysis of *C. arabica* Var. Typica Lines Used in Genetic Transformation

SEMs in plants can be the result of a natural (Garcês et al., 2007) or an artificial process (plant tissue culture medium), and it occurs when a somatic cell becomes into a totipotent embryonic stem cell to differentiate in an embryo with the potential to become a new plant. SEMs lack the development of an embryo sac, endosperm, and seed coat. SEMs play a critical role in clonal propagation, synthetic seed production, cryopreservation, genetic transformation, and genome editing.

In coffee, SEMs induced from leaf explants have been the most widely used target tissue in genetic transformation (Cortleven et al., 2019). SE induction and proliferation in coffee is time-consuming, ranging from 9 to 15 months until they can be used for stable genetic transformation (Barry-Etienne et al., 2002; Campos et al., 2017). In this work, we have induced a long-term induction and plant regeneration of SE lines of *C. arabica* var. Typica, previously used for stable transformation of coffee with a Bt toxin (Valencia-Lozano et al., 2019).

To understand the basic molecular mechanisms of SE induction in our embryogenic line, a set of genes derived from a network with a high confidence (0.700) performed in STRING (v11.0, http://string-db.org), based on *C. arabica* homologous genes present in *A. thaliana* genome, were evaluated.

The master regulators of SE, BBM, LEC1, and FUS3 were upregulated in our competent SE line (HC-SE) (5.0-, 5.9-, and 7.3-fold change, respectively) over the NC-SE line. Repression of complete SE competence was observed in double (*lec1 lec2, lec1 fus3*, and *lec2 fus3*) and triple (*fus3 lec1 lec2*) mutants in *Arabidopsis*. These mutants were able to regenerate plants *via* organogenesis derived from roots (Gaj et al., 2005). In cassava, *MeLEC1* and *MeLEC2* were highly upregulated in SEM cells in contrast with mature tissues. Increased levels were at early SE induction in the transition from somatic to embryogenic state (Brand et al., 2019). BBM promotes cell proliferation, differentiation, and morphogenesis, specifically during embryogenesis. BBM-induced embryogenesis relies on transcriptional activation of LEC1 and LEC2. LEC1 is a transcriptional activator required for both embryo maturation and cellular differentiation and FUS3, a regulator of gene expression during late embryogenesis (Horstman et al., 2017).

On the other hand, TANMEI/EMB2757 was 7-fold upregulated in our HC-SE line over the NC-SE line. It encodes a regulatory WD repeat protein required for both early and late phases of zygotic embryo development (Yamagishi et al., 2005) and SEM (Baster et al., 2009). In Arabidopsis, *tanmei/emb2757* (tan) mutation resulted in a total loss of embryogenic and organogenic capacity of cultured tissues, suggesting the involvement of TAN gene in basic cellular processes related to cell growth and differentiation. TANMEI/EMB2757 is a cell cycle checkpoint regulator, as it triggers the halt of cell cycle progression in the presence of DNA cross-linking agents.

Additionally, ETG1 and MCM4 genes were upregulated (9.3- and 10.3-fold) in the HC-SE line over the NC-SE one. They are involved in the cell cycle and maintenance of the genome. ETG1 binds to the MCM complex during late S phase and acts by promoting the disassembly of the MCM complex from chromatin. MCM4 double mutation in *Arabidopsis* impairs pollen development and is essential for embryo development (Long et al., 2019). MCM4 has a role in the mitotic cycle, consequently in cell proliferation, which is key for plant growth. Mutations induce different degrees of reduction in rosette size (González et al., 2020). ETG1 mutants are macroscopically normal, but an increase in cell size and endoreduplication occurs. Analysis of *etg1, tert*, and *mid* mutants was involved in DNA stress checkpoint activation indispensable for correct morphogenesis and survival (Cools and De Veylder, 2009).

Genetic transformation of plants can stall at several key points, including the acquisition of a stem cell-like state in cells as they re-enter the cell cycle, progression between the G1 and S phases of the cycle, and the differentiation of the transformed cell into a new embryo (Arias et al., 2006). Plant tissue culture medium (chemical formulation, plant growth regulators, and different stressing factors) and environmental signals (light and temperature) are essential for correct activation of the cell cycle during *in vitro* culture of plants. It will also depend on personal perception about plant growth regulators as the most important activators of differentiation of SEMs under *in vitro* conditions.

The upregulation of LEC1, FUS3, BBM, TANMEI/EMB2757, ETG1, and MCM4 in our HC-SE line reveals that the molecular mechanism for SE induction, maturation, and plant regeneration is partially due to the expression of these genes. Further experiments of transcriptomic and proteomic are required to elucidate these molecular mechanisms.

### Transgenic Coffee Fruits Resistant to CBB

Worldwide, *H. hampei* is the most damaging insect pest of coffee, causing an annual loss of around US$500 million. Global warming is driving CBB invasion and spread worldwide to warmer producer areas. CBB is the only insect pest of coffee that feeds and completes its life cycle in the coffee seeds (Johnson et al., 2020).

The major components of CBB integrated pest management (IPM) strategies used in most coffee-growing regions around the world include: monitoring of populations (traps and tree sampling), cultural control (preharvest and post-harvest sanitation), biological control (use of natural enemies), chemical control (use of insecticides and repellents), and physical control (exclusion netting and border crops) (Cabrera-Ponce et al., 2019).

Transgenic plants with insect resistance traits (IRTs) have contributed significantly to the agricultural industry on a commercial basis. The development of plants that undermine insect attack has been achieved by the expression of genes that encode toxins normally produced as Cry proteins in the soil bacterium Bt.

Genes for different Bt endotoxins are derived from assorted strains of the bacterium, and each insecticidal protein has different activity spectrum for various insect pests within the orders Lepidoptera and Coleoptera. As a toxic mechanism, Cry proteins bind to specific receptors on the membranes of mid-gut epithelial cells, resulting in the rupture of those cells. Any organism that lacks the appropriate gut receptors cannot be affected by the Cry proteins expressed in plants (Dorsch et al., 2002).

The toxic activity of Cry10Aa protein against coleopterans, such as CBB, when they tested individually all the Bti crystal components, except the Cry10A, and no toxicity was detected. The mortality of 52% was obtained using spore–crystal complex and 63% pure crystal with an estimated LC_50_ for the CBB of 219.5 ng/cm^2^ of diet.

This estimated LC_50_ is within the range reported for *Anthonomus grandis*, when using the same toxin transgenically expressed in cotton (Ribeiro et al., 2017). The importance of this work lies in the fact that it is the first time that the action of a coffee plant, transformed with the *cry10Aa* gene, shows efficient control of first instar larvae of CBB. Moreover, the 100% mortality shown by the two highest expression levels among the transformed events showed the great potential of these approaches to efficiently control this pernicious pest.

A successful protocol of genetic transformation of *C. arabica* var. Typica to integrate *cry10Aa* gene of Bt and be expressed was developed by Valencia-Lozano et al. (2019). These plants were grown in a greenhouse until fruit and seed sets were achieved. Different protein concentration was found in each transgenic line analyzed, ranged from 3.25 to 13.88 μg/g fresh weight. In cotton transgenic plants expressing Cry10Aa protein, a concentration of 6.35 μg/ml was found to be efficient to control *Anthonomus grandis* (Ribeiro et al., 2017).

As expected, variation of Cry10Aa expression was found in fruits of the T1 generation derived from three different events of transformation. Bioassays with first instar larvae and adults found that 75% of seeds was able to control CBB, yielding a 3:1 ratio, as a mendelian inheritance. This ratio was obtained on the T1 generation. Once homozygous lines are obtained, we expect a 90% efficiency or higher.

Evaluations made on fruits of transgenic plants revealed a direct effect on the infestation capacity of CBB, showing for the first time, the toxic effect of a Bt toxin on adults and larvae of an insect species, and an efficient control of *H. hampei*.

Physical damage shown by transgenic fruits varied from absolutely no damage to small holes in coffee seeds. Wild-type fruits showed that when challenged with one female CBB, they not only penetrated but they also built galleries for oviposition, reproduction, and larval feeding on the endosperm.

These results showed that the expression of the Cry10Aa protein in *C. arabica* plants is an efficient alternative to control CBB. It is important to notice that recombinant Cry10Aa protein has no apparent toxic effect in mice derived from genotoxic and hepatotoxic assays, highlighting its biosafety potential for use in transgenic crops (de Souza-Freire et al., 2014).

Transgenic plants evaluated in this work represent IRTs that potentially could have an impact on the environment by avoiding massive insecticide use and saving in carbon dioxide emissions (Brookes and Barfoot, 2016, 2017). In addition, these plants also have several agronomic traits that potentially can be used to resolve several problems in coffee farming (Valencia-Lozano et al., 2021).

## Conclusions

We demonstrated that the expression of Cry10Aa in elite embryogenic line allowed the transformed coffee plants to be toxic toward adults and larvae of *H. hampei*. This is the first report of coffee plants resistant to CBB.

Fruits of genetically transformed plants showed inhibition on the development and infestation capacity of *H. hampei* females, showing a successful and applicable control of this pest of global economic importance in *C. arabica*.

A significant improvement of SE resulted in solving important problems such as germination, time, and efficiency of genetic transformation.

## Data Availability Statement

The raw data supporting the conclusions of this article will be made available by the authors, without undue reservation.

## Author Contributions

EV-L conceived and designed the research, performed the experiments, analyzed the data, and wrote the manuscript. JC-P supervised the study, analyzed the data, and wrote the manuscript. JN-C involved in the supervision of greenhouse plants and analyzed the manuscript. JI conceived the research, supervision, analysis of data, and wrote the manuscript. All authors read and approved the manuscript.

## Conflict of Interest

The authors declare that the research was conducted in the absence of any commercial or financial relationships that could be construed as a potential conflict of interest.

## Publisher's Note

All claims expressed in this article are solely those of the authors and do not necessarily represent those of their affiliated organizations, or those of the publisher, the editors and the reviewers. Any product that may be evaluated in this article, or claim that may be made by its manufacturer, is not guaranteed or endorsed by the publisher.
